# Supplementary figures and images for: RhoGEF9 splice isoforms influence neuronal maturation and synapse formation downstream of α2 GABAA receptors
Source: PLoS Genet. 2017 Oct 25;13(10):e1007073. doi: 10.1371/journal.pgen.1007073 (PMC5673238; doi:10.1371/journal.pgen.1007073)

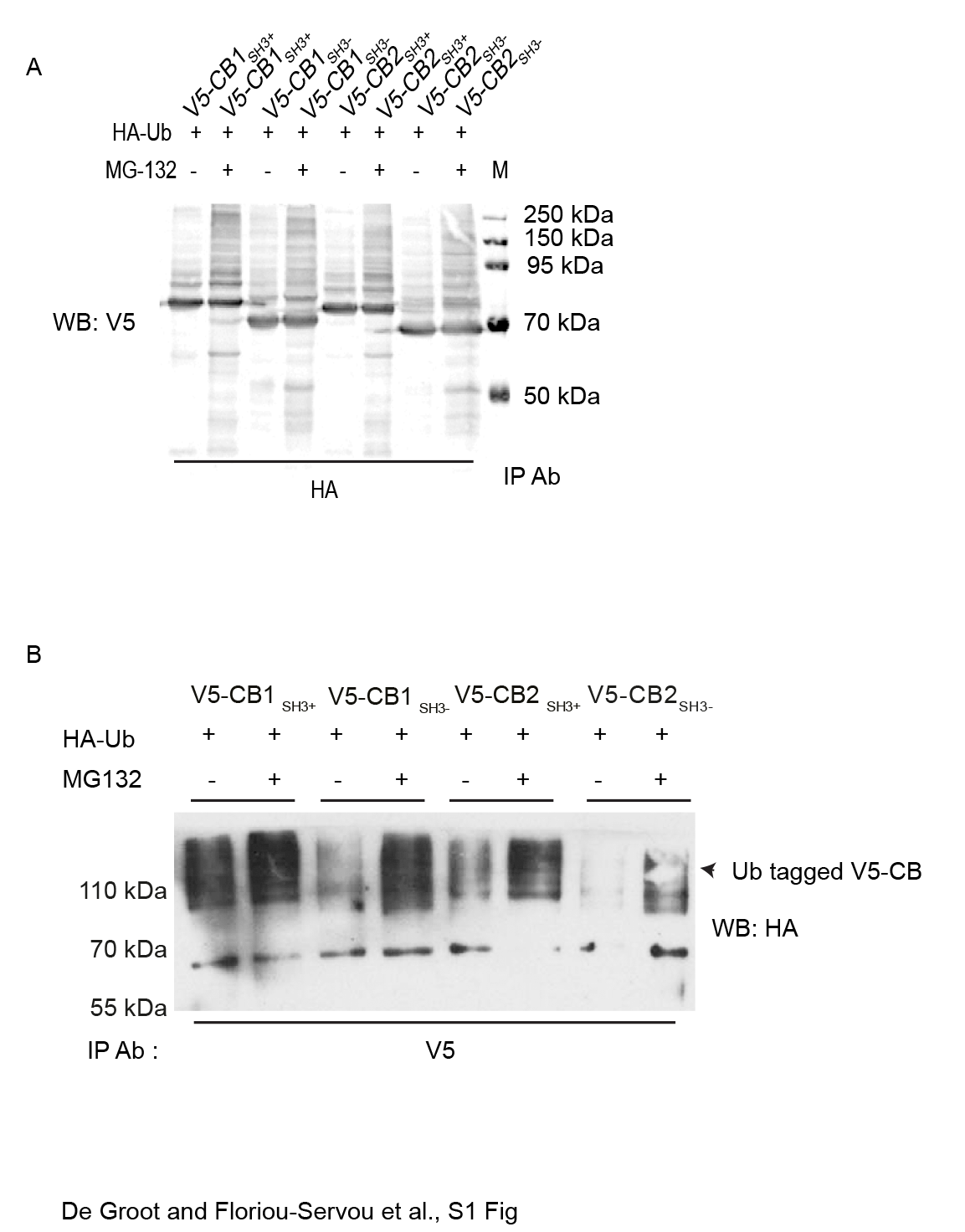

Supplement: S1 Fig — (A) HEK293T cells transfected with HA-Ubiquitin and V5-CB isoforms. The cells were treated with either DMSO or MG132 and analyzed for HA-Ub conjugation after IP for HA and WB for V5. Higher migrating V5 bands can be seen in MG132 treated samples. (B) HEK293T cells transfected with HA-Ub and V5-CB isoforms were treated with DMSO or MG132. IP for V5 followed by WB against HA-Ub showed enhanced Ub conjugated V5 CB isoforms in MG132 treated samples. (TIF) [file pgen.1007073.s001.tif]

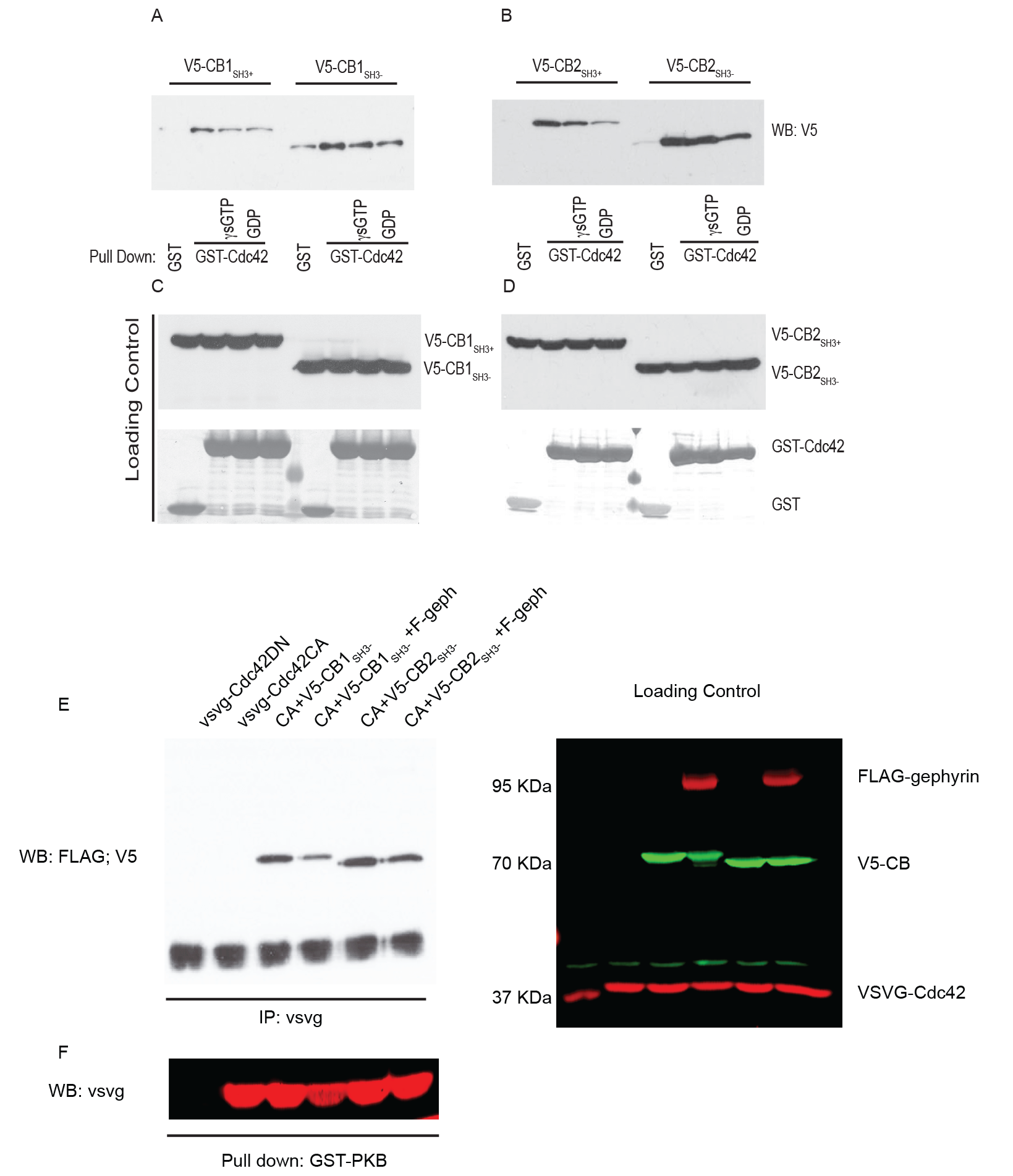

Supplement: S2 Fig — (A-B) Bacterial overexpressed and purified GST-Cdc42 incubated with γsGTP or GDP to mimic active and inactive forms. GST, GST-Cdc42, GST-Cdc42 γsGTP or GST-CDC42 GDP incubated with HEK293T lysates overexpressing V5-CB1SH3+, V5-CB1SH3-, V5-CB2SH3+ or V5-CB2SH3- showing interaction between active and inactive Cdc42 and CB isoforms. CB2 isoforms exhibit stronger interaction with Cdc42 compared to CB1 isoforms. (C-D) Protein loading controls showing equal amount of V5-CB isoform expression in HEK293T cells and GST-Cdc42 expression in bacteria. (E) HEK293T cells expressing vsvg-Cdc42 DN, vsvg-Cdc42 CA, vsvg-Cdc42 CA and V5-CB1SH3- or V5-CB2SH3- with or without FLAG-gephyrin. After pull down of free active Cdc42 from HEK293T cell lysate using GST-PKB we separated the supernatant for further analysis. We IP’ed vsvg-Cdc42 CA and performed WB against V5 to evaluate CB interaction with vsvg-Cdc42. Lanes containing FLAG-gephyrin co-expression showed reduced levels of CB interaction with vsvg-Cdc42 compared to CB alone lanes. (F) Pull down of free active vsvg-Cdc42 CA but not vsvg-Cdc42 DN using GST-PBD shows equal levels of active Cdc42CA in all lanes. Protein transfection levels in HEK293T cells are shown on the right panel. (TIF) [file pgen.1007073.s002.tif]

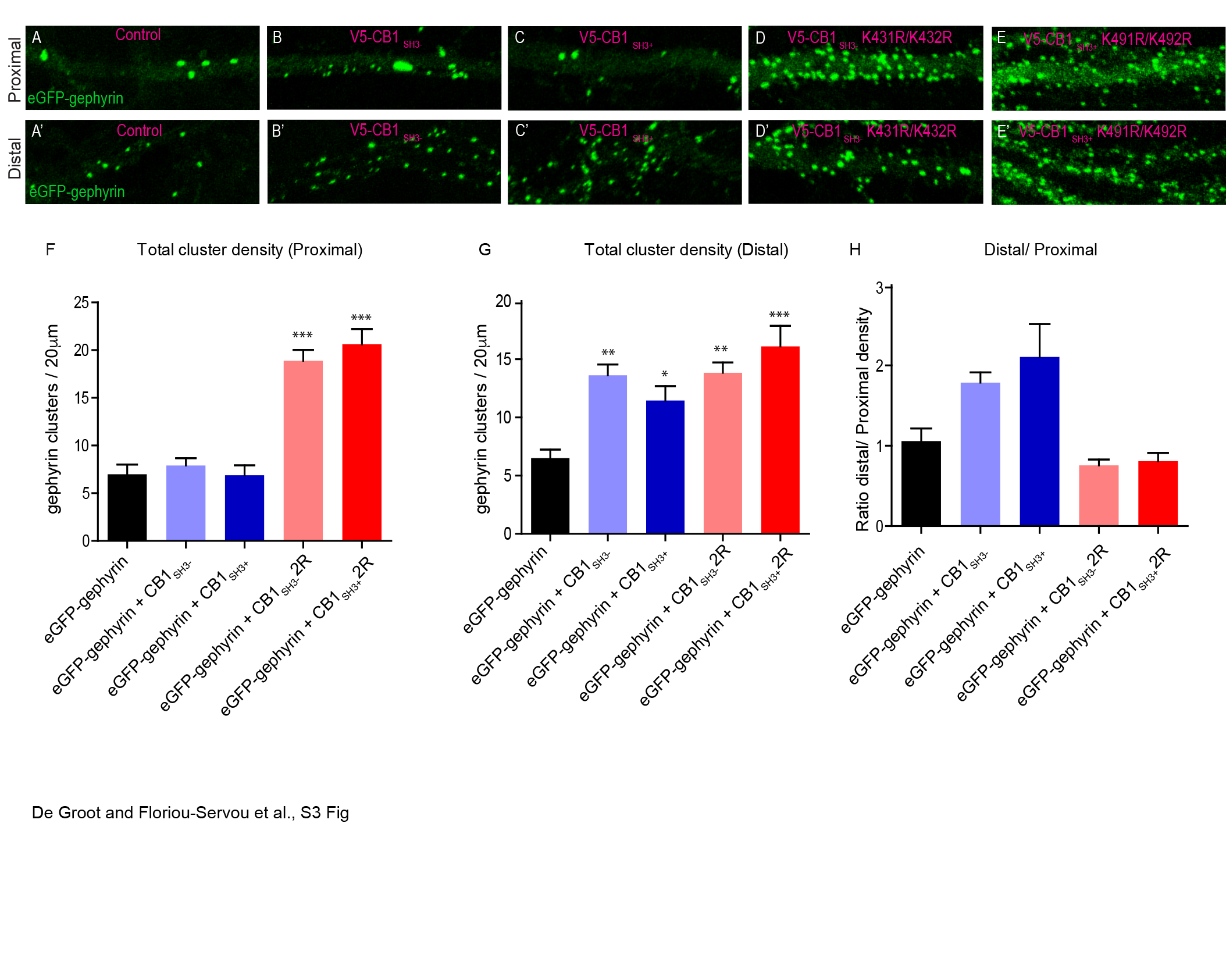

Supplement: S3 Fig — (A-E’) Morphological analysis of neurons co-transfected with eGFP-gephyin and CB1 ubiquitin mutants CB1SH3+ K491R/K492R or CB1SH3+ K491R/K492R. (F-H) Quantification of eGFP-gephyrin synaptic cluster density in neurons co-transfected with CB1SH3+ K491R/K492R or CB1SH3- K431R/K432R mutants show enhanced eGFP-gephyrin cluster density (DIV 8+7). Scale bar 5μm. One-way ANOVA with Kruskal-Wallis non-parametric test, Dunn's multiple comparison test p<0.0001). (TIF) [file pgen.1007073.s003.tif]

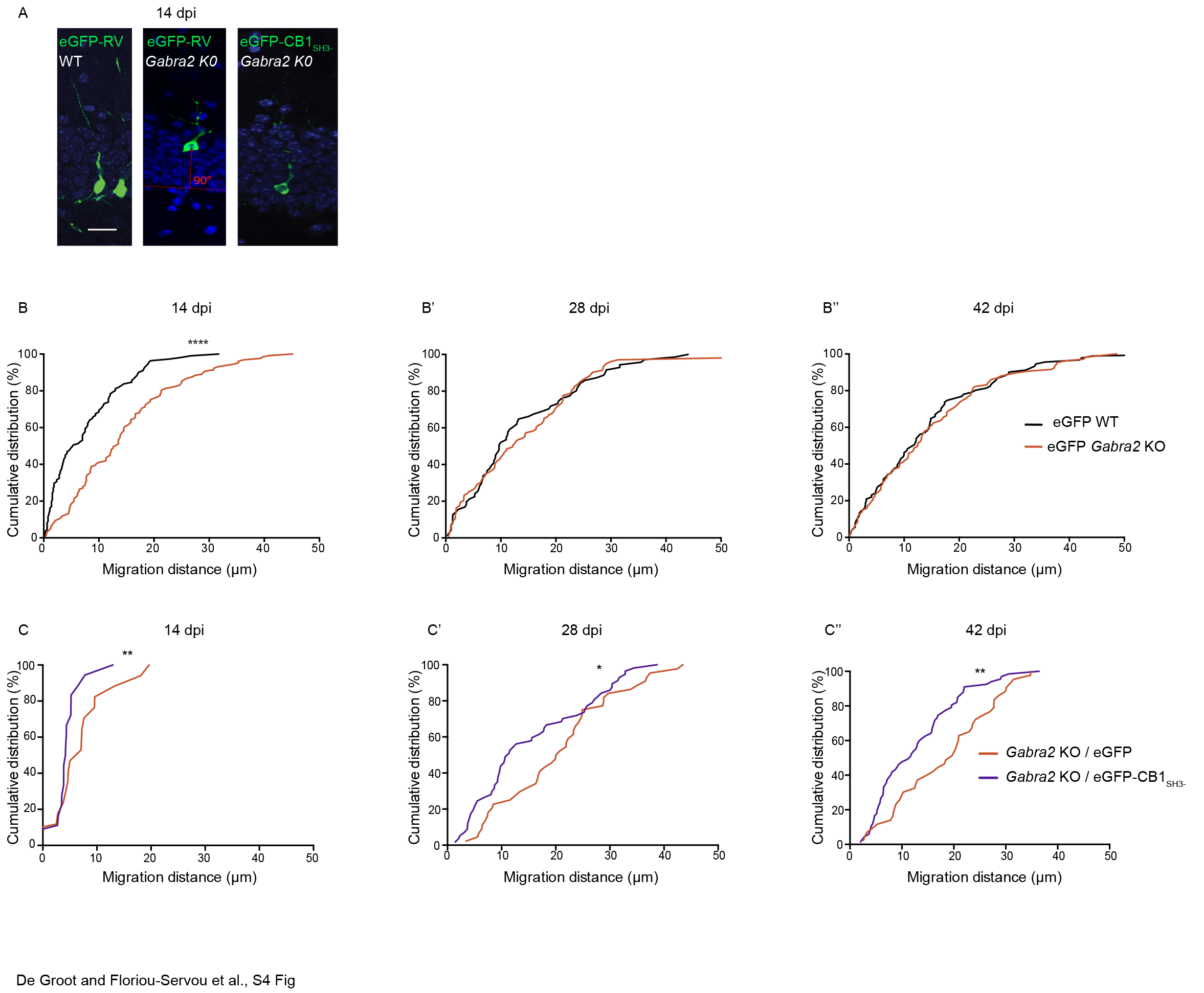

Supplement: S4 Fig — (A) Morphology of adult newborn neuron expressing eGFP in WT or Gabra2 KO background, and eGFP-CB1SH3- in Gabra2 KO background. (B-B”) Migration of adult newborn neurons in Gabra2 KO background is enhanced at 14 dpi compared to WT background. WT cells catch up at 28 dpi and 42 dpi. (C-C”) Comparison for neuronal migration in Gabra2 KO background show reduced migration in neurons expressing eGFP-CB1SH3- in comparison to eGFP control. (TIF) [file pgen.1007073.s004.tif]
